# Salivary alpha-amylase activity and cortisol in horses with acute abdominal disease: a pilot study

**DOI:** 10.1186/s12917-018-1482-4

**Published:** 2018-05-10

**Authors:** María Dolores Contreras-Aguilar, Damián Escribano, María Martín-Cuervo, Fernando Tecles, Jose Joaquín Cerón

**Affiliations:** 1 Interdisciplinary Laboratory of Clinical Analysis (Interlab-UMU), Veterinary School, Campus of Excellence Mare Nostrum, University of Murcia, 30100, Espinardo, Murcia, Spain; 2 Department of Food and Animal Science, School of Veterinary Medicine, Universitat Autònoma de Barcelona, 08193, Bellaterra, Barcelona, Spain; 3 Surgery Department, Faculty of Veterinary Medicine, University of Extremadura, 10005 Cáceres, Spain

**Keywords:** Salivary alpha-amylase, Salivary cortisol, Horse, Pain, Colic

## Abstract

**Background:**

The aim of this study was to evaluate salivary alpha-amylase (sAA), considered a non-invasive biomarker for sympathetic nervous system (SNS) activity, and salivary cortisol as possible pain-induced stress biomarker, in horses with acute abdominal disease. Therefore, a prospective observational study was performed in which both biomarkers were analyzed in a group of horses with acute abdomen syndrome, and compared with a group of healthy control horses by an unpaired Student’s t-test. In addition, the possible relationship between both biomarkers, the score in Equine Acute Abdominal Pain scales version 1 (EAAPS-1 scale), Heart Rate (HR) and Respiratory Rate (RR), plasma lactate, the systemic inflammatory response syndrome (SIRS) score and serum amyloid A (SAA) concentration was assessed by a Spearman correlation test.

**Results:**

A total of 30 horses were included in the study, 19 with acute abdominal disease diagnosed as large colon displacements, simple impactions of the pelvic flexure, spasmodic colics and enteritis and 11 healthy ones. sAA activity (24.5 median-fold, *P* <  0.0001) and salivary cortisol (1.7 median-fold, *P* <  0.01) were significantly higher in horses with acute abdomen than in healthy horses. sAA activity was significantly correlated with EAAPS-1 scale (*r* = 0.78, 95% confidence interval [CI] 0.38–0.89, *P* < 0.001) and SIRS score (*r* = 0.49, 95% CI 0.03–0.78, *P* < 0.05). Neither sAA nor salivary cortisol correlated with HR, RR, plasma lactate and SAA.

**Conclusions:**

Although this study should be considered as preliminary one, alpha-amylase measurements in saliva could be a biomarker of pain-induced stress in horses with acute abdominal disease.

**Electronic supplementary material:**

The online version of this article (10.1186/s12917-018-1482-4) contains supplementary material, which is available to authorized users.

## Background

Equine acute abdominal disease is one of the most important and relatively frequent diseases found in horses [1], being associated with different degrees of pain [2]. Currently, different scales have been developed for clinical use to evaluate the pain in the acute abdominal disease in horses such as the Equine Utrecht University Scale for Composite Pain Assessment (EQUUS-COMPASS) [1], the Equine Utrecht University Scale for Facial Assessment of Pain (EQUUS-FAP) [1], and the Equine Acute Abdominal Pain scales (EAAPS, version 1 and 2) [3]. In addition, physiological and endocrine measurements, such as heart (HR) and respiratory rate (RR), and blood catecholamines and cortisol levels have been used to assess pain in horses [4–6].

Saliva sampling is easy to obtain, non-invasive and less stressful than blood sampling [7]. Therefore, saliva would be an adequate sample for evaluating possible pain-induced stress level since reflects the activity of the sympathetic adrenal medullary system in individuals under stress. Plasma cortisol concentrations have a positive correlation with pain in horses [6] and a high correlation coefficient between blood and salivary cortisol concentrations has been found in horses [8]. Salivary alpha amylase (sAA) is considered as non-invasive biomarker for sympathetic nervous system (SNS) activity [9] and increases in psychological and physical stress situations [9, 10]. Previous reports have indicated the potential of sAA as an indirect marker for pain in mice [7]. In addition, sAA has been used in horses as a stress biomarker in different situations of physical efforts such as walking, trotting and dressage exercises [11]. So it could be postulated that measuring biomarkers like sAA or salivary cortisol can give a more reproducible method and objective assessment of pain-induced stress level in horses to contribute to an integrated description of state of pain. However, to the authors` knowledge, sAA activity and salivary cortisol have not been evaluated in equine acute abdominal disease.

The main objective of this study was to evaluate sAA activity and salivary cortisol in horses with acute abdominal disease as pain-induced stress biomarkers. For this purpose, a prospective observational study was designed in which sAA and salivary cortisol were analyzed in a group of horses with acute abdomen syndrome, and the possible changes in both biomarkers depending of the pain degree evaluated by the EAAPS-1 scale were studied. In addition, possible relationships between these salivary biomarkers, physiological variables such as HR and RR, the severity of the process evaluated by plasma lactate and the systemic inflammatory response syndrome (SIRS) score [12] and also the inflammation evaluated by serum amyloid A (SAA) concentration were assessed.

## Methods

### Stability pilot study

To evaluate sAA and salivary cortisol stability at room temperature and in refrigeration, a pilot study was performed. Saliva from five horses with different levels of sAA (ranging from 15.5 U/L to 59.1 U/L) and saliva cortisol (ranging from 0.17 μg/dL to 0.71 μg/dL) were selected. From each sample two aliquots were made: one was kept at room temperature (25 °C) and the second one was refrigerated (4 °C). Measurements were made just after saliva collection, 24 h and 48 h later.

### Clinical case and control population

The diseased population included private owned horses of two different provinces of Spain (Almería and Granada) showing abdominal pain that required the visit of a veterinarian and that were diagnosed as acute abdominal disease. Horses were evaluated by two veterinary specialists in equine clinic who visited the horses at their boxes. Information about age, gender, breed, treatment before presentation, heart rate, respiratory rate, pounding digital pulses and capillary refill time was collected. The clinical diagnosis was based on history, physical examination (abdominal auscultation, rectal examination and nasogastric intubation) and the results of additional diagnostics tests: (1) complete blood work by complete blood count (CBC) including hematocrit value (HCT), hemoglobin concentration, erythrocyte indices, platelet count, white blood cell (WBC) and differential WBC counts; (2) chemistry profile including plasma lactate and SAA; (3) abdominal ultrasound; (4) and abdominocentesis if it was necessary. Horses with pounding digital pulses or lameness that could indicate laminitis or synovitis [2] were not included in the study.

Eleven privately owned horses of the same provinces were selected as control group for being healthy based on history, physical examination, and a lack of abnormalities on CBC and serum biochemistry profile. Four females, two geldings and five stallions integrated this group, with a mean age of nine years (range of 2–15). The breeds included were six Spanish and five crossbred horses.

### Sampling

The samples collection was taken at the time of the veterinarian examination of the horse. In addition, sample collection was obtained before any potentially painful procedures (jugular venipuncture, nasogastric intubation, etc.).

Saliva samples were collected by introducing a small sponge in the horses’ mouth for at least 1 min, and they were placed in collection devices (Salivette, Sarstedt, Aktiengesellschaft & Co). Blood samples were collected after saliva sampling by jugular venipuncture. Saliva and blood were centrifuged at *4000 g* for 8 min at 4 °C and then transferred into 1.5 mL eppendorf tubes and stored at − 80 °C until analysis. Saliva and blood samples were referred in refrigeration to Interdisciplinary Laboratory of Clinical Analysis of UMU (*Interlab-UMU*) after 24–48 h. Horses that yielded serum samples with gross hemolysis and blood contaminated saliva samples were excluded from the study. No treatment was administered to the horses before the sample collection.

### Pain behavior scale

EAAPS-1 [3, 13] was evaluated in the habitual box of the horses with a previously history of abdominal pain by the veterinarians immediately upon arrival and before sample collection. This is based on a 5 point-score, which grades the severity of pain the horse is showing by picking the most severe behavior manifested, and the score for that particular behavior is the pain score (Table 1). Pain scale was translated to Spanish language and it was described in detail to the veterinarians before the beginning of the experimental study.

**Table 1:** Equine acute abdominal pain scale-version 1 (EAAPS-1)

Behaviors	Score
Depression	1
Flank watching	1
Weight shifting	2
Restlessness	2
Kicking abdomen	3
Pawing	3
Stretching	3
Sternal recumbence	3
Attempting to lie down	3
Lateral recumbence	4
Rolling	4
Collapse	5

To grade the severity of pain the horse is showing, pick the most severe behavior manifested, and the score for that particular behavior is the pain score. Table according to Sutton et al., (12)

### Assays for salivary biomarkers

sAA activity was measured using a colorimetric commercial kit (Alpha-Amylase, Beckman Coulter Inc.) following the International Medicine (IFCC) method [14], as previously reported and validated for horses [11], in an automatic analyser for biochemical assay (Olympus UA600, Olympus Diagnostica GmbH). It was expressed as U/L.

Salivary cortisol was analyzed with an immunoassay system (Immulite 1000, Siemens Healthcare Diagnostic), which uses a solid-phase competitive enzyme-amplified chemiluminescent immunoassay. The assay showed an intra-assay coefficient of variation lower than 15%, a parallel displacement to the standard linearly curve with serial sample dilutions and a analytical limit of detection of 0.016 μg/dL [15].

### Statistical analysis

The statistical analyses were calculated using Graph Pad Prism 6 (Graph Pad Software). sAA activity and salivary cortisol concentration results, EAAPS-1 score, HR and RR, plasma lactate concentration and SAA concentration were checked using Shapiro-Wilk test to assess normality, showing all of them a non-normal distribution except plasma lactate. Then, sAA and salivary cortisol results were transformed logarithmically by applying the formula ln x = ln (x + 1) [16] that restored normality before a unpaired Student’s t test (2-tailed) to determine if sAA and salivary cortisol were statistically different between clinical cases and controls.

The minimum number of individuals necessary to be included in each group for reaching a significance level of α = 5% (*P* < 0.05) and a power of 80% was calculated. For this purpose, means and standard deviations were initially calculated for the first 11 individuals received from the clinical cases and for the 11 control horses, and a stand-alone power programme for statistical testing commonly used in social and behavior research (G-Power) [17] was employed. This gave that a minimum number of seven individuals for each group get appropriate results for sAA and salivary cortisol. However, a post hoc analysis was performed with the final number of animals used in the study to guarantee that the significance level and power required were correctly obtained.

Additionally, a Spearman correlation between salivary biomarkers (sAA and salivary cortisol), pain scale, HR, RR, plasma lactate, the systemic inflammatory response syndrome (SIRS) score and SAA was performed. The SIRS is defined as having two or more abnormal results for any of the following: HR > 52 beats/min, RR > 20 breath /min, WBC above or below 5.0–12.5 × 10^9^/L, and temperature below or above 37.0–38.5 °C [12]. SIRS score is obtained on the number of abnormal SIRS criteria (4 point-score).

To study the stability of the salivary analytes measured, a repeated measures ANOVA and the Friedman post hoc test were used to evaluate changes in mean sAA and salivary cortisol in five saliva samples after storage at room temperature (25 °C) and refrigeration (4 °C) for 48 h. The limit for considering an acceptable stability for a given storage conditions was set as differences in values between time points lower than two intra-assay coefficient of variation (CV) of the assays (7.2% × 2 = 14.4% for sAA; and 12.5% × 2 = 25.1% for salivary cortisol) and no significant (*P* < 0.05) differences over time in relation to baselines levels (just after collection) [18].

## Results

### Clinical cases

Nineteen animals were included in the group of horses with acute abdominal pain, being seven females, four geldings and eight stallions. Average age was eight years (range of 1–19). The breeds included were nine Spanish horses, one Friesian horse, and nine were crossbred horses. Causes of the acute colic were diagnosed as large colon displacements (*n* = 10), simple impactions of the pelvic flexure (*n* = 5), spasmodic colics (*n* = 3) and enteritis (*n* = 1). All horses received medical treatment, since to the judgment of the clinicians it was considered more convenient. Of the 19 clinical cases, 17 survived while two horses with colon displacements died due to the disease. The two horses that did not survive showed the highest plasma lactate and SAA concentrations (one 18.08 mmol/L and 213.1 μg/mL, and the other 19.09 mmol/L and 308.5 μg/mL, respectively). Individual values about data required for the SIRS score calculation (HR, RR, temperature and WBC) in the disease horses are reported in the Additional file 1.

### sAA and salivary cortisol in acute colic

The five saliva samples used in the pilot stability study did not show significant changes in their sAA and salivary cortisol values at 24 and 48 h of storage compared with baseline values. In addition, the values obtained between the different time points did not differ more than two times the intra-assay CV.

**Fig. 1:**
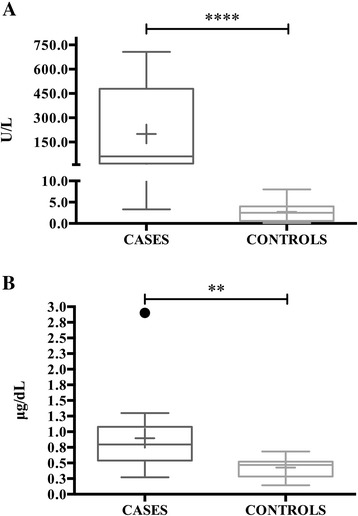
Salivary alpha-amylase (sAA) activity (**a**) and salivary cortisol (**b**) from horses with acute abdominal pain (cases) and healthy horses (controls). The plot shows median (line within box), 25th and 75th percentiles (box), 5th and 95th percentiles (whiskers) and outliers (•). The cross inside the box shows the mean. Asterisk indicates statistically significant difference (**P < 0.01; **** *P* < 0.0001) between groups

Median and quartiles values of sAA and cortisol appear in Fig. 1. Salivary cortisol was measured in 27 horses (16 of the acute abdomen group and 11 of the control group) due to insufficient saliva volume. sAA activity (U/L) in horses with acute abdomen was significantly higher than in healthy control horses (24.5 median-fold, *t*_25.44_ = 7.27, *P* < 0.0001). Salivary cortisol was also significantly higher in horses with acute colic than in healthy controls (1.7 median- fold, *t*_21.54_ = 3.43, *P* < 0.01). The highest values of sAA (706.7 U/L and 680.9 U/L) and salivary cortisol (2.9 μg/dL and 1.3 μg/dL) were presented in the two no-survivor horses. The post hoc analysis performed with the final number of individuals used in this study computed a power of 90% for the sAA results and 89% for salivary cortisol results.

### Correlation between parameters

Coefficients of correlation between salivary biomarkers and pain scale, HR, RR, plasma lactate, SIRS score, SAA and salivary flow rate are shown in Table 2. A significant correlation between sAA activity and pain scale was observed (*n* = 19, *r* = 0.78, 95% confidence interval [CI] 0.38–0.89, *P* < 0.001), while salivary cortisol did not correlate with pain scale (Table 2). In addition, sAA activity was also correlated with SIRS score (*n* = 19, *r* = 0.49, 95% CI 0.03–0.78, *P* < 0.05). Both salivary biomarkers did not correlate between them, neither with plasma lactate or SAA.

**Table 2:** Salivary alpha-amylase (sAA, *n* = 19) and salivary cortisol (*n* = 16) coefficients of correlation between Equine acute abdominal pain scales-version 1 (EAAPS-1), heart rate (HR) and respiratory rate (RR), plasma lactate, systemic inflammatory response syndrome (SIRS) score and Serum Amyloid A (SAA)

	sAA (U/L)	*P* values	Salivary cortisol (μg/dL)	*P* values
EAAPS-1 score	0.78 (0.38, 0.89)	< 0.001	0.11 (−0.42, 0.58)	0.685
HR (beats/min)	0.22 (−0.27, 0.62)	0.362	0.24 (−0.31, 0.67)	0.370
RR (breaths/min)	0.32 (−0.17, 0.68)	0.184	0.26 (−0.28, 0.68)	0.320
SAA (μg/mL)	0.29 (−0.21, 0.67)	0.235	−0.08 (− 0.54, 0.42)	0.761
Plasma lactate (mmol/L)	0.44 (−0.05, 0.76)	0.069	0.17 (−0.38, 0.64)	0.532
SIRS score	0.49 (0.03, 0.78)	0.032	0.16 (−0.38, 0.62)	0.560

Results are expressed as Spearman r-value (95% confidence interval [CI]). SIRS score (4 point-score) is based on the number of abnormal SIRS criteria among the following: HR > 52 beats/min, RR > 20 breath/min, WBC above or below 5.0–12.5 × 10^9^/L, and temperature below or above 37.0–38.5 °C

## Discussion

In veterinary practice, adequate diagnosis and treatment of painful conditions is dependent on accurate recognition of pain experienced by non-verbal animals [2], and represent an important way to improve the welfare and quality of care of the equine patient [19]. In horses, tools for objective assessment of pain such as cardiovascular measurements (HR and blood pressure) or biochemical determinations of plasma concentrations of β-endorphins, catecholamines and corticosteroids have been used for this purpose [1, 20]. In addition different scales based on external signs have been used to evaluate pain in horses; in this study EAAPS-1 scale was used because it is faster and easier to assess than EQUUS-COMPASS and EQUUS-FAP scales, being EAAPS-1 more reliable than EAAPS-2.

sAA activity was evaluated in our work as a potential indirect marker of pain showing a good correlation with EAAPS-1 and higher values in horses with acute abdominal disease compared to controls. Although comparisons should be made with caution due to the different species and nature of pain, the correlation found in our work was higher than those described in previous studies in humans such as heat pain perception [21], cancer with bone metastases [22] and chronic pain [23]. In addition, the magnitude of the increases of sAA found in colic cases compared to control group (24.5 median-fold) was higher than when pain was induced in mice (3.5 mean-fold) [7]. Overall, based in our results, it could be postulated that there is relation between pain and higher levels of sAA activity in horses with acute abdomen syndrome. Although further studies should be made to corroborate this, a possible cause of increasing of sAA would be the activation of SNS caused by pain [24]. No correlations were found between sAA and HR or RR. Although these parameters may be affected by pain and are often incorporated into composite pain scales as physiological parameters [2], several studies reported that, in general, physiological parameters are weakly associated with pain [4, 25, 26].

sAA was not correlated with plasma lactate and SAA, which are considered as markers of severity of disease [5] and acute inflammation in horses [27], as was moderately correlated with SIRS score. Overall this data would indicate that sAA is more linked with pain that with the inflammation that occurs in the cases of acute abdominal pain. Salivary cortisol did not correlate with pain scales in this preliminary study, although horses with colic showed higher values of cortisol than the healthy ones. However, previous reports showed a correlation between blood cortisol and a composite multifactorial pain scale (CPS) in acute orthopedic pain in horses [28]. Maybe the different nature of the pain stimulus could be the reason of this discrepancy. In addition, salivary cortisol did not correlate with sAA. The absence of correlation between sAA and salivary cortisol has been previously described [11, 29, 30]. One reason for this lack of correlation could be that sAA reflects the reaction of the SNS, while cortisol is related with the hypothalamic -pituitary-adrenal (HPA) axis [31, 32] having a different time of response, with the sAA release being faster than the cortisol. In all cases of our study, the colic were acute because they lasted lower than one day and the veterinarian attended the case as an emergency [33, 34], therefore it could be postulated that in some cases the cortisol could not reach its peak of concentration.

The pilot study made about sampling storage suggested that sAA activity and salivary cortisol were stable with the handling conditions of the saliva samples used in our study. Stability of the analyte is an important point when saliva is used as a sample since significant changes in concentrations can appear in some analytes under specific storage conditions, such as occurs with Norepinephrine in saliva after storage at 25 °C more than one hour [35].

This study has various limitations. One could be related with the accuracy of evaluation of the pain by the EAAPS-1 scale. Although EAAPS-1 was described in detail to the veterinarians before the study started and Sutton et al. [3] reported a good inter-rater reliability when scale was validated, it is important to point out that there are no standardized pain scale yet to assess the horses’ pain. Other limitation is that, although horses with possible laminitis or synovitis were excluded from the study, other possible pain diseases that could influence sAA values were not controlled, such as oral wounds [36]. In addition, the results of this study could not be extrapolated to situations of pain produced by other diseases such as laminitis or synovitis, and specific studies to evaluate the dynamics of both salivary biomarkers in pain conditions should be performed.

In our study, the time of sample collection varied since depended of when the veterinarian attended the emergency case. Although ideally, in order to avoid circadian rhythms, sampling should have been always made at the same time, this was not possible from a practical point of view since the priority was to explore and attend the horses as soon as possible. In any case, although there is no data in horses about circadian rhythms of sAA, if we consider the data reported in humans [37] with a maximum of 1.76 fold of increase from basal time at 9:00 to the peak which was found 16:00, this would not have a major influence in the differences obtained in our study, since median values in the clinical cases were 24.5-fold higher in healthy horses. Regarding the salivary cortisol, it has demonstrated an acrophase at 10:00 in horses [38] with a difference only of 0.03 μg/dL between 10:00 at 22:00 which is much lower than the median difference found in our study between the two groups of horses (0.3 μg/dL).

Although the statistical analysis confirmed that the number of horses used in our study was enough to evaluate differences between clinical and control horses, further studies involving a larger number of horses with acute abdominal disease would be recommended in order to confirm the relationship between sAA and pain. It is important to point out that pain evaluation do not replaces clinical decision making (which is made based on physical examination, blood results, ultrasound, and other diagnostic tests), but its objective evaluation by biomarkers could help to better evaluate the patient at admission and also to improve the follow-up during the treatment. In addition, it could have importance as a prognostic factor for the severity of colic [1]. Overall, this study should be expanded using a larger population to assess possible differences in the biomarkers depending of the origin of the acute abdominal disease and to evaluate the possible use of sAA and cortisol as a prognostic factor since the two highest sAA values were presented in the two no-survivor horses. In addition, it would be of interest to analyze a population of horses admitted to a hospital for non-painful, elective procedures to investigate the effect of other variables that cause stress for horses, such as transport and arrival to an unfamiliar facility, in the salivary biomarkers assessing in this study. Eventually, these studies could even lead to include sAA in future pain scales as a pain-induced stress biomarker.

## Conclusion

This study reports an increase of alpha-amylase activity in saliva of horses with acute abdominal disease, and it is correlated with EAPPS-1 pain scale. These preliminary results indicate that sAA could potentially be a biomarker of pain-induced stress in cases of horses with acute abdominal disease.

## Additional file

Additional file 1: Individuals values of heart rate (HR), respiratory rate (RR), white blood cell (WBC), temperature and systemic inflammatory response syndrome (SIRS) score in the disease horses (*n* = 19). Individuals values about data required for the SIRS score calculation (HR, RR, temperature and WBC) in the disease horses. SIRS state is defined as having two or more abnormal results for any of the following: HR > 52 beats/min, RR > 20 breath /min, WBC above or below 5.0–12.5 × 10^9^/L, and temperature below or above 37.0–38.5 °C. SIRS score is obtained on the number of abnormal SIRS criteria (4 point-score). non-SIRS: 0–1 abnormal criteria; SIRS2: 2 abnormal SIRS criteria; SIRS3/4: 3 or 4 abnormal SIRS criteria [12]. (XLSX 41 kb)

## Abbreviations

CBC: Complete blood work by complete blood count
CI: Confidence interval
CPS: Composite multifactorial pain scale
CV: Coefficient of variation
EAAPS-1: Equine Acute Abdominal Pain scales version 1
EQUUS-COMPASS: Equine Utrecht University Scale for Composite Pain Assessment
EQUUS-FAP: Equine Utrecht University Scale for Facial Assessment of Pain
HCT: Hematocrit
HPA: Hypothalamic -pituitary-adrenal axis
HR: Heart Rate
RR: Respiratory Rate
sAA: Salivary Alfa-amylase
SAA: Serum amyloid A
SIRS: Systemic inflammatory response syndrome
SNS: Sympathetic nervous system
WBC: White blood cell

## References

[1] Van Loon JPAM, VanDierendonck MC (2016). Monitoring acute equine visceral pain with the equine Utrecht University scale for composite pain assessment (EQUUS-COMPASS) and the equine Utrecht University scale for facial assessment of pain (EQUUS-FAP): a scale-construction study. Vet J.

[2] de Grauw JC, van Loon JPAM (2016). Systematic pain assessment in horses. Vet J.

[3] Sutton GA, Paltiel O, Soffer M, Turner D (2013). Validation of two behaviour-based pain scales for horses with acute colic. Vet J.

[4] Gleerup KB, Lindegaard C (2016). Recognition and quantification of pain in horses: a tutorial review. Equine Vet Educ.

[5] Hinchcliff KW, Rush BR, Farris JW (2005). Evaluation of plasma catecholamine and serum cortisol concentrations in horses with colic. JAVMA.

[6] Pritchett LC, Ulibarri C, Roberts MC, Schneider RK, Sellon DC (2003). Identification of potential physiological and behavioral indicators of postoperative pain in horses after exploratory celiotomy for colic. Appl Anim Behav Sci.

[7] Tsuchiya K (2014). Saidin MY bin, Inoue T, Kajiwara K, Kimura M. Qualitative measurement of pain by analysing the salivary alpha amylase. Precis Eng.

[8] Kedzierski W, Strzelec K, Anna C, Kowalik S (2013). Salivary cortisol concentration in exercised thoroughbred horses. J Equine Vet Sci.

[9] Nater UM, Rohleder N (2009). Salivary alpha-amylase as a non-invasive biomarker for the sympathetic nervous system: current state of research. Psychoneuroendocrinology.

[10] Granger DA, Kivlighan KT, El-Sheikh M, Gordis EB, Stroud LR (2007). Salivary alpha-amylase in biobehavioral research - recent developments and applications. Ann N Y Acad Sci.

[11] Fuentes-Rubio M, Fuentes F, Otal J, Quiles A, Tecles F, Cerón JJ (2015). Measurements of salivary alpha-amylase in horse: comparison of 2 different assays. J Vet Behav.

[12] Roy M, Kwong GPS, Lambert J, Massie S, Lockhart S (2017). Prognostic value and development of a scoring system in horses with systemic inflammatory response syndrome. J Vet Intern Med.

[13] Sutton GA, Dahan R, Turner D, Paltiel O (2013). A behaviour-based pain scale for horses with acute colic: scale construction. Vet J.

[14] van der Heiden C, Bais R, Gerhardt W, Lorentz K, Rosalki S (1999). IFCC methods for measurement of catalytic concentration of enzymes - part 9. IFCC method for alpha-amylase {[}1,4-alpha-D-glucan 4-glucanohydrolase, EC 3.2.1.1]. Clin Chim Acta.

[15] Escribano D, Fuentes-Rubio M, Ceron JJ (2012). Validation of an automated chemiluminescent immunoassay for salivary cortisol measurements in pigs. J Vet Diagn Investig.

[16] Rohleder N, Nater UM (2009). Determinants of salivary α-amylase in humans and methodological considerations. Psychoneuroendocrinology.

[17] Faul F, Erdfelder E, Lang A, Buchner A (2007). G*power: a flexible statistical power analysis program for the social, behavioral, and biomedical sciences. Behav Res Methods.

[18] Gröschl M, Wagner R, Rauh M, Dörr HG (2001). Stability of salivary steroids: the influences of storage, food and dental care. Steroids.

[19] VanDierendonck MC, van Loon JPAM (2016). Monitoring acute equine visceral pain with the equine Utrecht University scale for composite pain assessment (EQUUS-COMPASS) and the equine Utrecht University scale for facial assessment of pain (EQUUS-FAP): a validation study. Vet J.

[20] Valverde A, Gunkel CI (2005). Pain management in horses and farm animals. J Vet Emerg Crit Car.

[21] Wittwer A, Krummenacher P, La Marca R, Ehlert U (2016). Salivary alpha-amylase correlates with subjective heat pain perception. Pain Med.

[22] Hong L, Wen-yan D, Jian-bo W, Tao W, Peng H, Shu-fang W (2013). Association between salivary alpha-amylase activity and pain relief scale scores in cancer patients with bone metastases treated with radiotherapy. Chinese Med J.

[23] Shirasaki S, Fujii H, Takahashi M, Sato T, Ebina M, Noto Y (2007). Correlation between salivary alpha-amylase activity and pain scale in patients with chronic pain. Region Anesth Pain M.

[24] Mellor DJ, Stafford KJ, Todd SE, Lowe TE, Gregory NG, Bruce RA (2002). A comparison of catecholamine and cortisol responses of young lambs and calves to painful husbandry procedures. Aust Vet J.

[25] Price J, Catriona S, Welsh EM, Waran NK (2003). Preliminary evaluation of a behaviour-based system for assessment of post-operative pain in horses following arthroscopic surgery. Vet Anaesth Analg.

[26] Raekallio M, Taylor PM, Bennett RC (1997). Preliminary investigations of pain and analgesia assessment in horses administered phenylbutazone or placebo after arthroscopic surgery. Vet Surg.

[27] Dondi F, Lukacs RM, Gentilini F, Rinnovati R, Spadari A, Romagnoli N (2015). Serum amyloid a, haptoglobin, and ferritin in horses with colic: association with common clinicopathological variables and short-term outcome. Vet J.

[28] Bussieres G, Jacques C, Lainay O, Beauchamp G, Leblond A, Cadore J (2008). Development of a composite orthopaedic pain scale in horses. Res Vet Sci.

[29] Chatterton R, Vogelsong K, Lu Y, Ellman A, Hudgens G (1996). Salivary alpha-amylase as a measure of endogenous adrenergic activity. Clin Physiol.

[30] Nater UM, Rohleder N, Gaab J, Berger S, Jud A, Kirschbaum C (2005). Human salivary alpha-amylase reactivity in a psychosocial stress paradigm. Int J Psychophysiol.

[31] Engert V, Efanov SI, Duchesne A, Vogel S, Corbo V, Pruessner JC (2013). Differentiating anticipatory from reactive cortisol responses to psychosocial stress. Psychoneuroendocrinology.

[32] Sumter SR, Bokhorst CL, Miers AC, Van Pelt J, Westenberg PM (2010). Age and puberty differences in stress responses during a public speaking task: do adolescents grow more sensitive to social evaluation?. Psychoneuroendocrinology.

[33] Mair TS, Edwards GB (2007). Chronic and recurrent colic: challenging clinical syndromes. Equine Vet Educ.

[34] Mair TS, Hillyer MH (1997). Chronic colic in the mature horse: a retrospective review of 106 cases. Equine Vet J.

[35] Kennedy B, Dillon E, Mills PJ, Ziegler MG (2001). Catecholamines in human saliva. Life Sci.

[36] Ashley F, Waterman-Pearson A, Whay H (2005). Behavioural assessment of pain in horses and donkeys: application to clinical practice and future studies. Equine Vet J.

[37] Rohleder N, Wolf JM, Maldonado EF, Kirschbaum C (2006). The psychosocial stress-induced increase in salivary alpha-amylase is independent of saliva flow rate. Psychophysiology.

[38] Bohák Z, Szabó F, Beckers JF, Melo de Sousa N, Kutasi O, Nagy K (2013). Monitoring the circadian rhythm of serum and salivary cortisol concentrations in the horse. Domest Anim Endocrin.

